# Single-lipid tracking reveals heterogeneities in the nanoscale dynamics of colloid-supported lipid bilayers†

**DOI:** 10.1039/d4sm01299b

**Published:** 2025-03-26

**Authors:** Levena Gascoigne, Roderick P. Tas, Pepijn G. Moerman, Ilja K. Voets

**Affiliations:** a Laboratory of Self-Organizing Soft Matter, Department of Chemical Engineering and Chemistry, Eindhoven University of Technology P.O. Box 513 5600 MB Eindhoven The Netherlands i.voets@tue.nl R.P.Tas@tudelft.nl; b Institute for Complex Molecular Systems (ICMS), Eindhoven University of Technology P.O. Box 513 5600 MB Eindhoven The Netherlands; c Department of BioMechanical Engineering, Delft University of Technology, Mekelweg 2 2628CD Delft The Netherlands

## Abstract

In this work, we utilize single-particle tracking photoactivated localization microscopy (sptPALM) to explore lipid dynamics in colloid-supported lipid bilayers (CSLBs) with liquid-like (DOPC), gel-like (DPPC), and phase-separated (DOPC:DPPC:cholesterol) membranes. Using total internal reflection fluorescence illumination, we tracked photoactivatable fluorescent dyes conjugated to lipids within these membranes. Analysis of tracked lipids revealed that bilayers across all compositions have heterogeneous dynamics, with lipid mobility varying over three orders of magnitude. We leveraged the temperature-dependent phase behavior of DPPC to transform gel-like membranes at room temperature into liquid-like membranes above 41 °C, which resulted in increased diffusivity and a surprising decrease in heterogeneity. Finally, we perform single lipid tracking in fluid-rich phases within gel-phase regions to demonstrate their dynamics with reduced lipid mobility because of soft confinement within phase-separated microdomains. Our findings have implications for colloidal assembly strategies that exploit ligand mobility to create controlled and reproducible colloidal superstructures.

## Abstract 2

Single-particle tracking photoactivated localization microscopy was used to explore lipid dynamics in colloid-supported lipid bilayers, highlighting the inherent heterogeneity in lipid mobilities and their implications for self-assembly applications.

## Graphical abstract

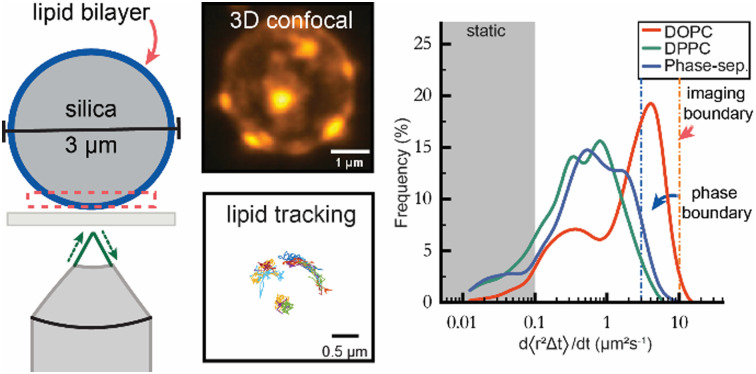

## Introduction

Colloid-supported lipid bilayers (CSLBs) are appealing membrane models and versatile building blocks for dynamic colloidal systems. CSLBs composed of multiple lipids have increased our understanding of curvature-depended phase separation^1^ and multivalent binding events.^2,3^ Additionally, CSLBs have been used to create dynamic colloidal superstructures, termed colloidal molecules, which resemble real molecules by incorporating surface-mobile DNA linkers within the bilayer.^4–7^ The application of CSLBs as colloidal molecules has been significantly aided by advances in our fundamental understanding of their bilayer structure and lipid dynamics. Creating colloidal molecules with tailored structures and flexible joints requires the lipid membrane to be fluid and homogeneous.^4^ However, the fluidity and homogeneity of lipid membranes have been investigated in-depth on the micron scale,^4,8^ and nanoscale heterogeneities, that could locally affect membrane fluidity and mobility, may have gone undetected.

The fluidity of CSLBs is typically examined using fluorescence recovery after photobleaching (FRAP) microscopy.^4,8^ This diffraction-limited method provides valuable insights into the ensemble-averaged behavior of lipids within the membrane.^4,5,8–11^ However, to investigate nanoscale lipid dynamics and reveal local variations in lipid composition and organization, single lipid resolution needs to be achieved. Single-particle tracking photoactivated localization microscopy, sptPALM, is a direct, super-resolution approach to access the short time scales (∼ms) and diffraction-unlimited length (∼nm) scales associated with the diffusion of single (macro)molecules in complex systems.^12^ In this technique, single fluorophores are conjugated to target molecules and stochastically activated to determine their location with nanometric accuracy from a Gaussian fit to their point spread function. Subsequently, the mobility of individual targets, *e.g.* lipid diffusion within membranes, is determined by tracking the resolved positions corresponding at high framerate to achieve both high spatial and temporal resolution. sptPALM has previously been applied to reveal and quantify the dynamics of key components of (sub)cellular membranes with high spatiotemporal resolution,^12–15^ offering unprecedented insight into the behavior of individual lipids and proteins in real-time.^14–17^ Additionally, in planar supported lipid bilayers as model membranes, pioneering studies have revealed nanoscale heterogeneity^16^ and identified dynamic differences in the mobility of individual lipids residing in the distal or proximal leaflets of the membranes.^14,15,17^ However, the extent to which local membrane heterogeneity and individual lipid dynamics change within CSLBs remains elusive.

In this work, we aimed to resolve the dynamics of lipids within colloid-supported lipid bilayers with sptPALM. To this end, we prepared CSLBs with liquid-like (DOPC), gel-like (DPPC), and lipid-phase separated (DOPC:DPPC:cholesterol) membranes on 3 μm silica colloids. Using total internal reflection fluorescence (TIRF) illumination, we studied the dynamics of photoactivatable fluorescent dyes conjugated to lipids incorporated within the membrane. Intriguingly, quantitative analysis reveals heterogeneities within all colloid-supported bilayer compositions. Exploiting the temperature-dependent phase behavior of DPPC, we transformed gel-like membranes at room temperature into liquid-like membranes at temperatures above 41 °C. With increasing temperature, the mean diffusivity increased, and the heterogeneity decreased. Finally, we explore the potential of sptPALM to identify heterogeneities in both the structure and dynamics of CSLBs using a multicomponent lipid bilayer that can undergo phase separation. We achieved high-resolution images of fluid-rich phases amidst gel-phase regions, revealing decreased lipid mobility due to phase separation-induced confinement. This work underscores the versatility and promise of sptPALM in investigating the dynamics of complex colloid-supported membrane systems. We expect that this technique can be used to explore the intricate behavior of functionalized CSLBs, including phenomena such as additive-induced membrane clustering, insights into the kinetics of fluid DNA multivalent interactions, and determining differences in inner and outer leaflet lipids mobilities.

## Experimental section

### Materials

1,2-Dioleoyl-*sn-glycero*-3-phosphocholine (DOPC), 2-dipalmitoyl-*sn-glycero*-3-phosphocholine (DPPC) and cholesterol were purchased from Avanti lipids *via* Sigma Aldrich. Additionally, 1,2-dioleoyl-*sn-glycero*-3-phosphoethanolamine-*N*-(CAGE 590) (DOPE–CAGE_590_) and 1,2-dipalmitoyl-*sn-glycero*-3-phosphoethanolamine-*N*-(CAGE 590) (DPPE–CAGE_590_) used for imaging where obtained from Abberior GmbH. All solvents and salts (HEPES, PBS, CaCl_2_, MgCl_2_, NaCl, sucrose, chloroform) used for liposome hydration were purchased from Sigma Aldrich. ∅ 3.0 ± 0.14 μm (SiO_2_-R-L3628) silica particles were obtained from Microparticles GmbH.

### Preparation of small unilamellar vesicles

Small unilamellar vesicles (SUVs) made of 1,2-dioleoyl-*sn-glycero*-3-phosphocholine (DOPC) or 1,2-dipalmitoyl-*sn-glycero*-3-phosphocholine (DPPC), or DOPC, DPPC, and cholesterol (phase-separated) were formed *via* thin-film method. Briefly, main lipid stock solutions (10 mM) of DOPC, DPPC and cholesterol of were suspended in chloroform. Stock lipids were added to 15 mL PTFE falcon tubes for a final lipid concentration of 1 mM for lipid film creation. For phase-separated SUVs, ratios of DOPC : DPPC : cholesterol were 30 : 60 : 10 molar %, respectively. This ratio was chosen to induce lipid phase separation, resulting in distinct liquid-ordered and gel-phase regions.^18^ To provide fluorescence to the bilayer, a small amount of dye-conjugated phospholipid, with phase behavior similar to the primary lipid, was included in the lipid mixture. Specifically, for single lipid tracking 2.5 nM of 1,2-dioleoyl-*sn-glycero*-3-phosphoethanolamine-*N*-(CAGE 590) (DOPE–CAGE_590_) was incorporated into DOPC and phase-separated films, and 2.5 nM 1,2-dipalmitoyl-*sn-glycero*-3-phosphoethanolamine-*N*-(CAGE 590) (DPPE–CAGE_590_) incorporated into DPPC. For confocal fluorescence recovery after photobleaching measurements, dye concentrations were increased to 25 nM lipid–CAGE_590_.

The chloroform was evaporated using a stream of N_2_ while vortex mixing, until a dry (by eye) lipid film remained. The lipid film was placed under a vacuum overnight at room temperature and put in the freezer until required (within 1 month). Hydration of single-phase lipid films was done using 10 mM HEPES and 50 mM NaCl buffer at pH 7.4 above the melting temperature of the primary lipid (DOPC; 20 °C, DPPC; 60 °C) with no extrusion. Hydration of phase-separated films was completed with PBS, 1 mM CaCl_2_, 0.5 mM MgCl_2_ and 200 mM sucrose buffer pH 7.4 at 60 °C with no extrusion. We found a PBS sucrose buffer to result in the best chance of rafted bilayers using passive lipid film hydration. In contrast, the HEPES did not perform consistently in achieving the latter. After mixing for 2 minutes, samples were sonicated for 30 minutes and then added directly onto 3 μm silica particles (see below).

### Sample preparation of colloid-supported lipid bilayers

Colloid-supported lipid bilayers (CSLBs) were formed *via* the mixing and deposition of SUVs (200 μL, 1 mM) onto 3 μm silica particles (30 μL, 50 mg mL^−1^) in a solution of buffer (940 μL, hydration buffer). Samples were mixed rapidly (stirrer bar in glass 5 mL vial) at 300 rpm above the melting temperature of the primary lipid (DOPC; 20 °C, DPPC; 60 °C, phase-separated system; 60 °C). After 1 hour, samples were washed *via* centrifugation 5 times in room temperature solution buffer (10 mM HEPES and 50 mM NaCl buffer at pH 7.4). After washing CSLBs were stored at 4 °C. Samples were imaged within 2 days, as previous FRAP experiments^8^ shown a substantial decrease in lipid diffusion after 2 weeks in storage due to lipid oxidation. Additionally, to mitigate the effect of lipid oxidation, we avoided repeatedly cycling samples from 4 °C to room temperature and back.

For imaging, a sample channel was made by placing a #1.5 coverslip (Thermo Scientific Menzel ×1000, #1.5, 22 × 22 mm) with double-sided tape (Scotch Double Sided Tape 665) at the edges on to the center of a microscopy cover slide (VWR microscope slides 631-1552, borosilicate 76 × 26 mm). Both cover slip and cover slide were washed in three rounds of sonication in Milli-Q water followed by sonication in isopropanol and dried by stream of N_2_. 20 μL CSLB samples were injected into the sample cell and sealed with nail varnish, allowing the sample to sediment. The use of double-sided tape creates a gap in the viewing chamber greater than 3 μm, ensuring that the bilayer at the top of the CSLB is suspended freely in bulk and does not come into contact with the glass. In contrast, at the bottom of the chamber, the bilayer is predicted to be approximately 17 nm from the glass slide, due to the formation of a trapped water layer between the outer leaflet and the silica support.^19^

### Sample preparation of planar supported lipid bilayers

Planar supported lipid bilayers (PSLBs) were formed *via* the deposition of SUVs (30 μL, 1 mM) into the sample channels as stated above. SUV samples were heated above the melting temperature of the primary lipid (DOPC; 20 °C, DPPC; 60 °C, phase-separated system; 60 °C) before deposition and placed in a preheated oven at the same temperature for approximately 20 minutes. After 20 minutes, samples were washed by slowly rinsing the slide with 2 mL of buffer (10 mM HEPES and 50 mM NaCl buffer at pH 7.4), sealed with nail varnish, and analyzed immediately. Due to the thickness of the double-sided tape, the gap of the viewing chamber ensures the bilayer at the top (outer leaflet) of PSLB is free in bulk, whereas at the bottom of the viewing chamber, the bilayer (inner leaflet) is predicted to be approximately 17 nm away from the slide due to formation of a trapped water layer between outer leaflet and silica support.^19^

### Single-particle tracking photoactivated localization microscopy

Single-particle tracking photoactivated localization microscopy (sptPALM) was performed on a Nikon Eclipse Ti-E N-STORM, TIRF system equipped with a Nikon 100× Apo TIRF oil immersion objective (NA 1.49) and perfect focus system. Excitation was performed with the 640 nm laser within the MLC400B laser box (Agilent technologies) under TIRF or HiLo illumination through a quad-band polychroic mirror (Nikon 97335). An Ixon3 EMCCD (Andor) was used for detection, resulting in an effective pixel size of 160 nanometer. The microscope was equipped with a custom temperature control system for the temperature-controlled phase transition experiments in the DPPC samples.^20^ Samples were mounted and imaged with exposure times of 10 ms, respectively.

### Single molecular localization and track generation

sptPALM acquisitions were imported in ImageJ/Fiji^21^ and analyzed using the detection of molecules (DoM version 1.2.5, https://github.com/ekatrukha/DoM_Utrecht) plugin. A signal-to-noise ratio between 3.5 and 5 was chosen to detect single molecules. For single particle tracking, the ‘link particles to tracks’ function was used with a maximum permitted distance of 3 or 2 pixels from the next detected position and a maximum linking gap of 3 or 2 frames for DOPC and DPPC, and phase-separated containing CSLBs, respectively.

### Calculation of mean square displacement and *D*_eff_ from lipid trajectories

Exported DoM files were imported into a custom Python script for analysis. We considered tracks with a minimal track length of 10 points for analysis and calculated the 2D time-ensembled-averaged measured square displacement (MSD) as a function of delay time, denoted as 〈*r*^2^(Δ*t*)〉:^22^

M1 〈*r*^2^(Δ*t*)〉 = 〈[*r*(*t* + Δ*t*) − *r*(*t*)]^2^〉

where *r*(*t*) represents the position of the particle in *X*,*Y*, at time *t*, and *r*(*t* + Δ*t*) represents its position at a time lag Δ*t* later. The MSD of a Brownian trajectory with Gaussian noise in the particle detection is given by;^22^

M2 〈*r*^2^(Δ*t*)〉 = 2*σ*^2^ + 4*D*Δ*t*

where *σ* is particle positional error, and 4*D* is the diffusion coefficient. The standard practice in obtaining the diffusion coefficient is to perform a linear fit to MSD, where the slope is the effective diffusion coefficient (*D*_eff_), and the *Y*-intercept is 2*σ*^2^. Due to limitations in track lengths, we report the results of *D*_eff_ from the linear fitting of the first two data points of the MSD, giving an approximate relative error of 41% for track lengths of 10 points. See ESI,† Section 1 for further detailed explanation and results.

## Results

### sptPALM experimental overview

**Fig. 1:**
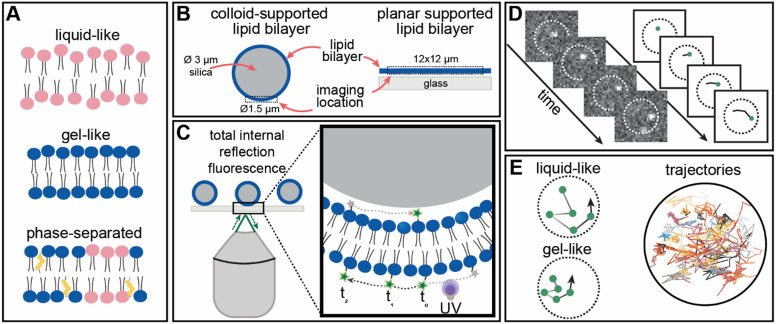
(A) Schematic of lipid ordering in liquid-like, gel-like, and phase-separated membranes. (B) Schematic representation of 3 μm colloid- and planar supported lipid bilayer samples used. (C) Total internal reflection fluorescence microscopy setup. Individual lipid-dye molecules are switched ‘on’ using UV irradiation, and fluorescence over time is recorded. (D) Single-molecule tracking workflow. Over time, a series of frames (left) is acquired to localize individual fluorophores. The trajectories of the lipids are reconstructed from the positions of tracked dye molecules over time (right). (E) Examples of expected single-molecule trajectories in liquid-like and gel-like regions within the membrane and a random selection of single-molecule trajectories from a single CSLB DOPC sample.

To study the organization and dynamics of lipids in CSLBs with high spatiotemporal resolution, we prepared supported bilayers with DOPC (liquid-like), DPPC (gel-like), and DOPC:DPPC:cholesterol (phase-separated) membranes on 3 μm (diameter) silica colloids and glass slides (Fig. 1A and B). We prepared lipid membranes with a small fraction (0.0025 mol%) of DOPE or DPPE conjugated to caged photoactivatable fluorescent dyes (–CAGE_590_) (Fig. 1C). This low labeling efficiency combined with UV-controlled conversion of the caged fluorophore allowed sequential visualization of single lipid trajectories in the membranes (Fig. 1D). Subsequent detection and localization of the nanometric position of each lipid over time resulted in high-accuracy tracking using sptPALM (Fig. 1E).

**Fig. 2:**
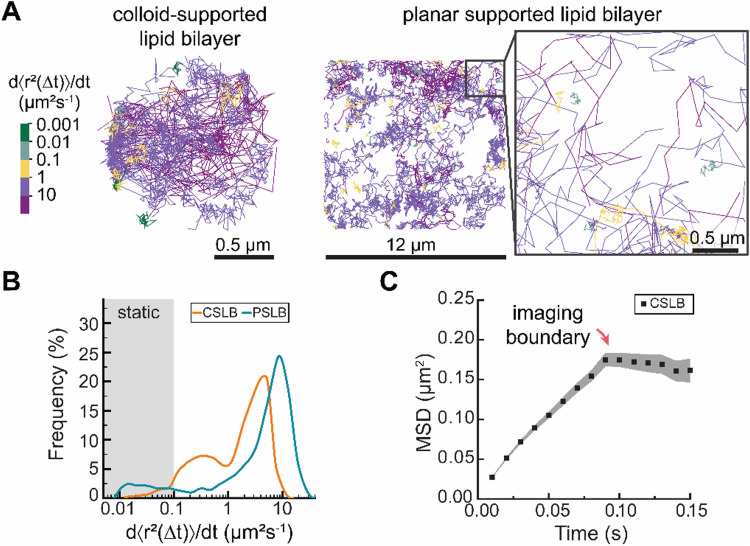
(A) Randomly selected examples of lipid trajectories at room temperature for colloid-supported and planar supported DOPC bilayer samples. Trajectories are color-coded based on the corresponding mobility (d〈*r*^2^(Δ*t*)〉/d*t*). (B) Distribution of the mobility of lipids within the colloid- (orange, 514 tracks) and planar supported (blue, 2473 tracks) bilayers with a minimum track length of 10 frames (100 ms). The distribution histogram is shown as an Akima Spline connected line plot. (C) MSD of CSLBs with plateau region indicating 3D imaging boundary.

**Fig. 3:**
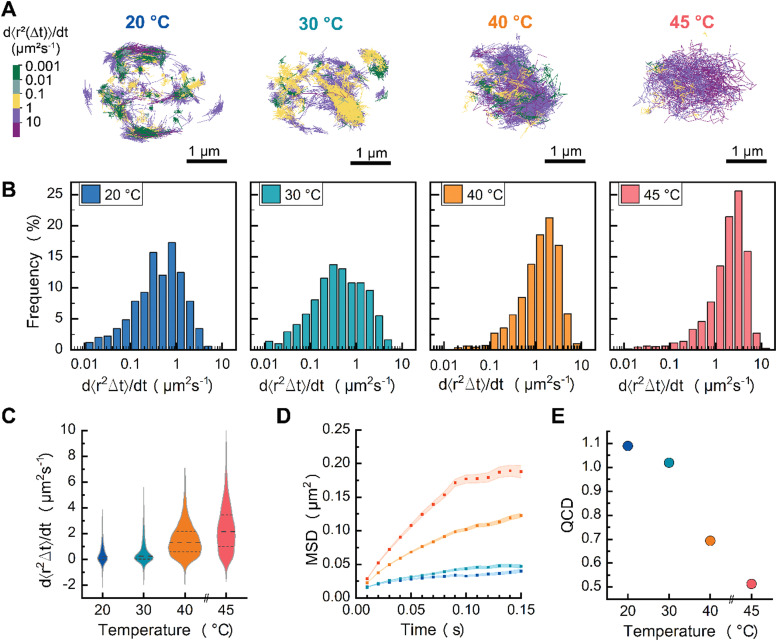
(A) Particle trajectories of DPPC (melting temperature of 41 °C) at 20, 30, 40 and 45 °C. (B) Log histogram of effective diffusion coefficient distributions and (C) violin plot of the effective diffusion coefficient distributions of DPPC CSLBs as a function of temperature. (D) MSD of DPPC CSLBs as a function of temperature. (E) Quartile coefficient of dispersion (QCD) of effective diffusion coefficient within samples.

**Fig. 4:**
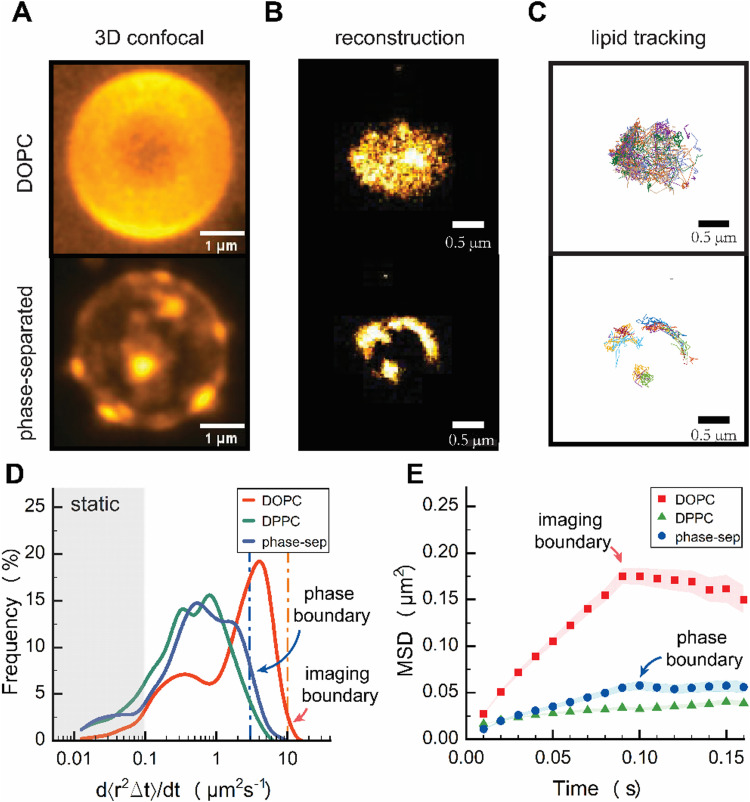
(A) Maximum intensity projection of Z-stack of DOPC (top) and phase-separated (bottom) CSLBs using conventional confocal microscopy and (B) TIRF microscopy images from the reconstruction of PALM localizations. (C) Single-lipid particle trajectories from sptPALM (D) distribution of effective diffusion coefficient of DOPC and phase-separated CSLB at 20 °C (E) ensemble-averaged MSD for DOPC, DPPC, and phase-separated CSLB tracks at 20 °C. Error bars are from standard error.

We first assessed the feasibility of sptPALM on liquid-like DOPC CSLBs and compared them to planar supported bilayers (Fig. 2). Next, we conducted variable temperature experiments on DPPC CSLBs to probe lipid dynamics below and above the melting temperature (Fig. 3). Finally, we explored the potential of sptPALM to identify heterogeneities in both the structure and dynamics of CSLBs by using membranes that could undergo phase separation (Fig. 4).

### Tracking single-lipids reveals heterogeneity in the dynamics of liquid-like colloid-supported lipid membranes

To evaluate the performance of sptPALM in CSLBs, we investigated the dynamics of DOPE–CAGE_590_ lipids within apparently homogeneous liquid-like membranes of both colloid- and planar supported lipid bilayers (PSLBs) (Fig. 2). We prepared these liquid-like membranes by assembling small unilamellar vesicles (SUVs) from hydrated lipid films containing DOPC and DOPE–CAGE_590_ mixed in a 4 × 10^5^ : 1 molar ratio. We rapidly mixed the SUV solution with 3 μm silica colloids or statically incubated the solution on a microscopy slide to form CSLBs and PSLBs, respectively.

The transient UV-mediated conversion of the caged dyes led to a sparse population of activated fluorophores, enabling the localization of hundreds of individual DOPE lipids for 0.1–5 seconds per trajectory (Experimental section). For each trajectory, we calculated the time-averaged MSD and quantified their mobility with d〈*r*^2^〉/d*t*, which we obtained from fitting the first two non-zero data points to avoid underestimating the mobility due to 3D confinement (see ESI† for an in-depth account of the method).^23^ We estimated that under these illumination conditions the effect of the particle's curvature on the projected trajectories was minimal; the angle between the imaging plane and the sphere's tangent never exceeds 10 degrees.

Color-coding individual lipid tracks based on the effective diffusion rate (*D*_eff_) revealed substantial heterogeneity in the dynamics of individual lipid trajectories in both CSLBs and PSLBs (Fig. 2A). The distributions of the lipid mobilities in CSLBs and PSLBs spanned about three orders of magnitude, with lipids in CLSBs diffusing slower than in PSLBs (Fig. 2B). We found a median diffusion coefficient of 2.1 μm^2^ s^−1^ and 5.2 μm^2^ s^−1^ for CSLBs and PSLBs, respectively. The mean diffusion coefficients of PLSB lipids are in close agreement with previously reported values ranging 4.4–4.9 μm^2^ s^−1^.^14,17^

The degree of heterogeneity in the diffusion of single lipids (including static lipids, which have *D*_eff_ < 0.1 μm^2^ s^−1^) was similar between the two samples; the quartile coefficient of dispersion (QCD) of 0.83 for CSLBs and 0.86 for PSLBs. If we exclude static lipids from the QCD (7.7% of the total PSLB population and <0.01% for CSLB), the QCD was 0.76 for CSLBs and 0.52 for PSLB, suggesting that PSLBs have a larger fraction of static lipids than CSLBs but that the mobility of the mobile lipids in PSLBs is more narrowly distributed. We had expected the distribution of *D*_eff_ to be similar for both CSLB and PSLB, however, CSLBs appear far more heterogeneous than compared to PSLBs.

Further, we expected the upper limit of *D*_eff_ of lipids in CSLBs and PSLBs of the same lipid composition to be similar, however it appears that the *D*_eff_ of lipids within PSLBs are 2-fold larger than CSLBs. Tentatively, we attribute this difference to a more pronounced hindrance of the diffusive motion of lipids in the exterior leaflet of bilayers on colloid supports compared to planar supports. A small subset of lipids in the exterior leaflet of CSLBs is in direct contact with the glass slide (onto which the colloid has settled), effectively sandwiching these lipids between a planar support on one side and the interior leaflet on a colloid support on the other. Additionally, stronger lipid–substrate interactions, arising from differences in surface chemistry (CSLB: SiO_2_, PSLB: borosilicate), may further contribute to this effect. However important to note is that the *D*_eff_ of lipids is likely an underestimate for CLSBs because our analysis is biased to slower lipids that stay longer in the imaging plane (SI 1, ESI†). Imaging on a spherical surface imposed an upper limit on CSLB sptPALM. Lipids with *D*_eff_ > 10 μm^2^ s^−1^ moved out of imaging plain before we could obtain sufficient track length for analysis, resulting in a weak inverse correlation between track length and *D*_eff_ (Fig. S2, ESI†). This upper limit resulted in underestimating mean *D*_eff_, making CSLB lipids appear slower and causing a confinement plateau in the MSD (Fig. 2C).

The cause of lipid diffusion heterogeneity remains contested,^24^ as the diffusion constants vary depending on the method used to detect these. Researchers attribute membrane diffusion heterogeneity to differences in friction of the lipid heads in the interior leaflet (monolayer-substrate coupling) and exterior leaflets (monolayer-fluid coupling),^14,25,26^ and to local variations in bilayer composition^27^ and/or structure, such as nanoscale surface defects.^28^ The absence of localized spots of slow or fast diffusing lipids suggests that the observed heterogeneity in lipid mobility is not due to surface impurities or confinement in the contact zone (where the CSLB and imaging slide contact) but rather an innate lipid bilayer property. Schoch *et al.*^14^ observe a bimodal distribution in the lipid mobility of PSLBs that is consistent with the range of mobilities that we observe in Fig. 2B and assigned high mobilities to lipids in the outer leaflets and low mobilities to lipids in the inner leaflet of the bilayer. We speculate that the heterogeneity of lipid diffusion in the supported membranes studied herein at least partially results from the differences in the friction of lipids in the interior and exterior leaflets. Selective incorporation of DOPE–CAGE_590_ into interior or exterior leaflets would make it possible measure whether lipids in the inner and outer leaflet have differing mobilities.

Taken together, our measurements indicate that CSLBs exhibit more heterogeneous lipid dynamics compared to PSLBs, which contain a larger fraction of static lipids. Notably, the slower dynamics observed in CSLBs are (partially) attributed to the upper detection limit imposed by imaging on a spherical surface.

### Temperature-induced reduction in lipid ordering increases the average mobility and reduces the dynamic heterogeneity in DPPC membranes

Now that we have shown that we can measure distributions of individual lipid mobilities with sptPALM, we wanted to investigate how these distributions change as the bilayers undergo a phase transition from a gel-like to a liquid-like state. Specifically, we addressed whether there are two populations, one with high and one with low mobility, of which the relative abundance changes, or if the whole population shifts from high to low mobility. Previous DSC experiments demonstrated that DPPC-based supported lipid bilayers (on 320 nm glass beads) displayed homogeneous gel-like behavior at room temperature and melted around 39–41 °C (*T*_m_), with no identifiable pre-transition state.^29^ To directly visualize this gel-to-liquid phase transition with single lipid precision, we prepared DPPC CSLBs with DPPE–CAGE_590_ mixed in a 400 : 0.001 molar ratio and performed sptPALM (Fig. 3). To control the temperature of the samples, we mounted CSLBs on a Peltier-controlled stage and heated at 3 degrees per minute.^20^

Color-coding individual trajectories showed that the average mobility of DPPC lipids increased with rising temperature (Fig. 3A). We find that the DPPC dynamics were heterogenous at all studied temperatures with *D*_eff_ spanning three orders of magnitude at each measured temperature (Fig. 3B). With increasing temperature, we observed that the lipids’ mobilities increased (Fig. 3C). Importantly, none of *D*_eff_ distributions exhibited a clear bimodal pattern, indicating the absence of distinct populations of gel-like and liquid-like lipids. This absence of separate groups implies that the transition from gel-like to liquid-like states involves cooperative changes within the bilayer. In other words, as some lipids shift their orientation or packing, they influence neighboring lipids to adjust similarly, resulting in a synchronized transformation across the bilayer structure.

At temperatures below *T*_m_, the lipids were, on average, less mobile than those measured in the liquid-like DOPC bilayer, suggesting they were in the gel-like phase. Above *T*_m_, we noted a plateau region similar to that observed for DOPC, indicating that the lipids transitioned to a liquid-like state (Fig. 3D). The substantial spread in lipid diffusion at temperatures below *T*_m_ hints at interesting lipid dynamics that are rarely resolved experimentally, revealing that a DPPC bilayer in a gel-like state still contains high-mobility lipids. Previously reported molecular dynamics simulations provide potential explanations for this dynamic behavior in gel-like membranes.^30^ Shafique *et al.*^30^ demonstrated that, at temperatures below *T*_m_, DPPC membranes exhibit two distinct lipid mobility subpopulations: slow-moving lipids, which are slightly more ordered and trapped by their neighbors in a gel-like state, and a small fraction of mobile lipids with low local orientational order. Additional atomic force microscopy experiments have shown the coexistence of gel-like and liquid-like lipid regions in DPPC membranes during melting and cooling ramps.^31^

In terms of heterogeneity, we characterized the lipid mobilities using the coefficient of dispersion (QCD), which decreased with increasing temperature (Fig. 3E). Notably, at temperatures below *T*_m_, the QCD of DPPC was greater than that of DOPC (QCD of 0.83). However, above *T*_m_, the QCD of DPPC lipids was less than that of DOPC, indicating that DPPC lipids in a fluid state exhibit a less heterogeneous diffusion distribution compared to DOPC lipids in the same state. These results suggest that DPPC CSLBs at 40 °C could offer better uniformity in lipid mobility than DOPC at room temperature. For researchers aiming to have homogeneity lipid dispersion with consistent diffusion speeds, heated DPPC CSLBs may be a preferred option. This could be particularly useful in systems requiring uniform lipid dynamics, such as multi-ligand binding or lateral diffusion of DNA patches. At elevated temperatures, the increased lipid fluidity reduces heterogeneity in diffusion, ensuring that functionalized groups—whether DNA patches or colloidal joints—are not constrained by slower-moving lipids. This could facilitate more predictable interactions, improve binding efficiency, and enhance the overall responsiveness of lipid-anchored functional groups.

In summary, we demonstrated the applicability of sptPALM to monitor the impact of temperature on lipid membrane dynamics across a range of temperatures—below, near, and above the *T*_m_—facilitating the transition from gel-like to liquid-like phases. Our findings indicate that the *D*_eff_ of DPPC lipids increase, while dynamical heterogeneity decreases with rising temperature. Notably, the dynamics of *D*_eff_ shift from low to high mobility with increasing temperature, rather than exhibiting distinct populations of gel-like and fluid-like lipids. Further experimental exploration of dynamic heterogeneity in DPPC CSLBs, utilizing MINFLUX microscopy for enhanced spatial and temporal resolution, would provide valuable insights into these dynamics.

### Heterogeneity of lipid dynamics in phase-separated domains

Finally, to test to what extent we could resolve local diffusion rates using sptPALM, we created spatially heterogeneous colloid-supported bilayers from DOPC:DPPC:cholesterol membranes that partially phase-separated into two or more coexisting domains with different local lipid compositions.^32^ We prepared DOPC, DPPC, cholesterol, and DOPE–CAGE_590_ SUVs in a 3 : 6 : 1 : 0.05 molar ratio for confocal and a 12 : 24 : 4 : 0.001 molar ratio for TIRF (Fig. 4).

3D confocal imaging of phase-separated CSLBs showed distinct submicron-sized patches consistent with liquid-like domains surrounded by gel-like phase (Fig. 4A and Fig. S6, ESI†). The overall patch sizes were heterogeneous on the colloid and within samples, with patch sizes seemingly about 500 nm in width. Next, we reconstructed images of DOPE–CAGE_590_ within a homogeneous DOPC, and phase-separated CSLBs from the individual lipid localizations obtained in TIRF (Fig. 4B). Similar to the 3D confocal images, the reconstructed TIRF images revealed bright patches of high-density DOPE–CAGE_590_ lipids in the phase-separated CSLBs and the improved diffraction-unlimited resolution now clearly revealed patch sizes even below 500 nm in width (Fig. 4B). Subsequent analysis of individually reconstructed tracks (Fig. 4C) also displayed a heterogeneous distribution of diffusion rates among phase-separated lipids (Fig. 4D).

We anticipated DOPE–CAGE_590_ to be within a fluid-rich domain;^33^ however, the effective diffusion coefficient distribution shown in Fig. 4D surprisingly displayed a distribution that resembled a gel-like phase domain. We expect that small effective diffusion constants were due to the underestimation of lipid mobility resulting from our analysis, which involved fitting the first two data points from the MSD. As shown in Fig. 4E, the initial slope of phase-separated dynamics (until 0.1 s) was substantially different from DPPC, indicating that they behave different and are not in a DPPC-rich phase. However, the individual lipids are definitely not as mobile as pure DOPC systems. Additional FRAP experiments (Fig. S7, ESI†) suggested that the DOPE–CAGE_590_ preferentially partitioned into fluid regions due to showing partial signs of recovery, whereas no recovery is measured for gel-like systems.^8^

We attribute the decrease in DOPE–CAGE_590_ mobility to a high density of DOPC lipids within the confined area; DOPE–CAGE_590_ experiences local crowding due to soft confinement within the microphase-separated domains. Clear signs of confinement were evident in the ensemble-averaged MSD analysis, with a plateau at 0.05 μm MSD, corresponding to a confinement phase boundary of ∅ 250 nm (Fig. 4E). Furthermore, at the 30 : 60 : 10 molar ratio of DOPC : DPPC : cholesterol, the presence of coexisting liquid-disordered and liquid-ordered domains is plausible. This could cause lipids within the liquid-ordered regions to exhibit slower-than-expected mobilities due to the increased packing density and restricted diffusion in these domains.^34^

Our results show that lipids confined within fluid patches in gel regions exhibit slower dynamics than those in single-phase CSLBs, primarily due to soft confinement effects.

## Conclusion

sptPALM provides a complementary approach to FRAP, enhancing our understanding of lipid mobilities in complex colloid-supported lipid bilayer systems. By utilizing sptPALM on colloid-supported lipid bilayers, we successfully resolved individual lipid dynamics, revealing a surprising degree of local membrane heterogeneity and individual lipid behavior, independent of composition (DOPC or DPPC). Notably, at 40 °C, DPPC CSLBs exhibit more uniform distribution in lipid mobility than DOPC, positioning them as a preferable option for researchers seeking consistent diffusion rates in applications reliant on uniform lipid dynamics.

Crucially, our findings indicate that the unexpected variability in individual lipid mobilities, regardless of lipid composition, carries significant implications for researchers utilizing functional mobile lipids in self-assembly applications. A thorough understanding of this heterogeneity is essential, as it may influence the reproducibility and reliability of experimental results.

While the exact origins of the observed lipid diffusion heterogeneities remain unclear, it is evident that lipid diffusion on colloid supports is inherently heterogeneous. To investigate whether inner leaflet lipids interacting with the substrate contribute to this heterogeneity, we propose using sptPALM on CSLBs with polymer cushions inserted between the substrate and lipid interface to minimize lipid–substrate interactions. Furthermore, the dynamical heterogeneity observed in this study could inform colloidal assembly strategies that leverage homogeneous ligand mobility, ultimately facilitating the creation of designer colloidal superstructures and functional colloidal materials in a controlled and reproducible manner.

## Author contributions

LG, RPT, and IKV conceived and designed the study. LG & RPT designed and performed the experiments. LG & RPT analyzed the data with input from IKV. LG, RT, PM, and IKV interpreted the results. IKV and RPT supervised the research. LG, RPT, PM, and IKV wrote the manuscript.

## Data availability

The data supporting this article have been included as part of the ESI.†

## Conflicts of interest

There are no conflicts to declare.

## Supplementary Material

SM-021-D4SM01299B-s001

SM-021-D4SM01299B-s002
