# Supplementary material for: Single-lipid tracking reveals heterogeneities in the nanoscale dynamics of colloid-supported lipid bilayers
Source: Soft Matter. 2025 Mar 26;21(16):3058–66. doi: 10.1039/d4sm01299b (PMC11957375; doi:10.1039/d4sm01299b)
Supplement: SM-021-D4SM01299B-s002 [file SM-021-D4SM01299B-s002.pdf]

Supplementary Information (SI) for Soft Matter.
This journal is © The Royal Society of Chemistry 2025

Single-lipid tracking reveals heterogeneities in the nanoscale dynamics of colloid-supported lipid bilayers: supporting information

Levena Gascoigne[1#], Roderick P Tas [1,2#]*, Pepijn G Moerman[1], Ilja K. Voets[1*]

#Equal contribution
**Corresponding authors*
[1]Laboratory of Self-Organizing Soft Matter, Department of Chemical Engineering and Chemistry, Eindhoven University of Technology, P.O. Box 513, 5600 MB Eindhoven, The Netherlands
Institute for Complex Molecular Systems (ICMS), Eindhoven University of Technology, P.O. Box 513, 5600 MB Eindhoven, The Netherlands

[2]Department of BioMechanical Engineering, Delft University of Technology, Mekelweg 2, 2628CD Delft, The Netherlands

Table of contents

page

1 **Calculating MSD, effective diffusion coefficient, and optimal track length for single particle tracking** 1

Figure S1 Distribution of the length of tracked lipids in DOPC colloid-supported (left) and planar-supported (right). 1

Table S1 Calculated relative error associated with taking short track lengths (N < 50) and taking τ points of 2 or 3 (m) 1

Figure S2. A) Distribution histogram of colloid and planar-supported DOPE-lipids d$\langle r^2 \rangle$/dt with minimum track length cuff-off of 10,15 and 20. **B)** Box Distribution of colloid (left) and planar (right) supported DOPE-lipids d$\langle r^2 \rangle$/dt with minimum track length cuff off of 10,15 and 20. With p<0.05=* and p<0.001=***. **C)** Correlation between interpolated d$\langle r^2 \rangle$/dt values and track length of colloid (left) and planar (right) supported DOPE-lipids d$\langle r^2 \rangle$/dt 2

Figure S3. A) Distribution of colloid and planar-supported DOPC bilayer interpolated data from d$\langle r^2 \rangle$/dt with varying the minimum number of points ($P_{start-end}$) with varying initial offsets (1,2,3) from the first non-zero data point. **B)** The Distribution of extrapolated particle positional error ($2s^2$) with P_{1-2} and P_{2-3} for colloid and planar-supported the DOPC bilayer. The minimum number of points in a track is 10, with N=539 for CSLB and N= 2841 3

Table S2 Dunns multiple comparisons for CSLB and PSLB with a minimum track length of 10 4

2 **Quartile Coefficient of Dispersion** 4

3 **RAW sptPALM particle tracks, MSD, and D_{eff} distribution of DOPC CSLBs** 5

Figure S4. DOPC CSLBs all samples, with all **A)** measured tracks blue Deff < 1.78, red Deff > 1.78 μm2/s, **B)** MSD, and C) histogram of individual Deff. 5

Figure S5. DOPC PSLBs all samples, with all measured tracks blue Deff < 1.78, red Deff > 1.78 μm2/s 6

4 **Confocal imaging and FRAP analysis** 6

Figure S6. Maximum Z projection images of **A)** DOPC, **B)** DPPC, and **C)** phase-separated CSLBs. 7

Figure S7. A) Examples of (left) z max projection taken of particles prior to bleaching, the (middle) bottom of particles prior to bleaching with ROI bleached, and (right) after bleaching **B)** FRAP recovery curve of DOPC, DPPC, and phase-separated CSLBs 8

5 **References** 8

1. Calculating MSD, effective diffusion coefficient, and optimal track length for single particle tracking

The mean square displacement (MSD) for all particles was computed according to Equation S1.

$$\langle \mathrm{r}^2(\Delta t)\rangle = \langle [r(t+\Delta t) - r(t)]^2\rangle \qquad \text{Equation S1}$$

where r(t) represents the position of the particle in X,Y, at time t, and r(t+Δt) represents its position at a time lag Δt later. The MSD of a Brownian trajectory with Gaussian noise in the particle detection is given by;

$$\langle \mathrm{r}^2(\Delta t)\rangle = 2\sigma^2 + 4D\Delta t \qquad \text{Equation S2}$$

where s is particle positional error, and *D* is the diffusion coefficient. The standard practice in obtaining the diffusion coefficient is to perform a linear fit to $\langle r^2\rangle$, where the slope is D, and the Y-intercept is $2s^2$. An estimate of the relative error on D is given by Equation S3; [1]

$$\frac{\delta D_m}{D_m} \sim \left[\frac{2m}{3(N-m)}\right]^{1/2} \qquad \text{Equation S3}$$

where m is the last point considered in the linear curve, and N is the total number of points in the trajectory. We explored the optimal analysis conditions using DOPC-tracked lipids on planar and colloid substrates to maintain a low relative error while maintaining key information.

We find that most track lengths (N) are below 50 points for colloid-supported and 100 for planar-supported (Fig. S1). This is well below the ideal situation, where ideally, track lengths should be >130 to ensure a relative error ~10%. We did not want to exclude shorter track lengths (<100), specifically for CSLB, as these short trajectories are influenced by imaging on a spherical substrate (Fig. SI.2). We, therefore, explored the relative error associated with taking short track lengths (N < 50) and taking either Δt points of 2 or 3 for the linear fitting to find D and report it in Table S1.

From Table S1, we decided on an acceptable relative error between 27—40 %. We applied a linear fitting to colloid and planar DOPC-tracked lipids with a minimum of 10, 15, and 20 points in the track length to see if there were any significant differences in the distribution mean and variance in interpolated slope values (Fig. S2).

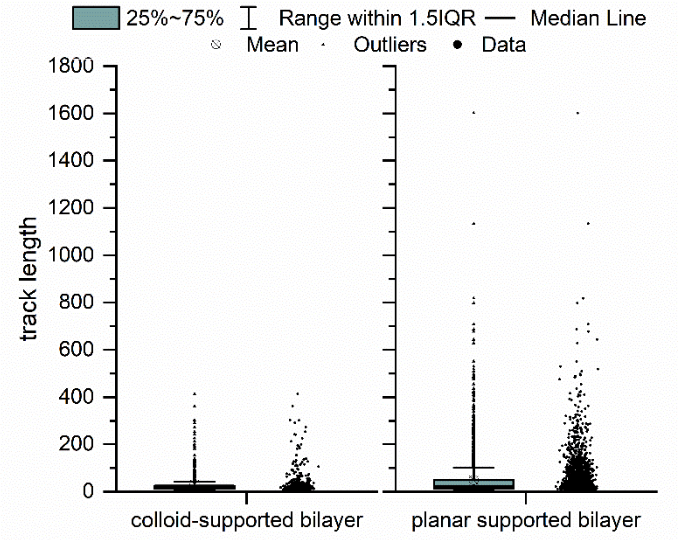

Figure S1 Distribution of the length of tracked lipids in DOPC colloid-supported (left) and planar supported (right).

Table S1 Calculated relative error associated with taking short track lengths (N < 50) and taking τ points of 2 or 3 (m)

N_{min} / m	50	20	15	10	5
2	17%	27%	32%	41%	67%
3	21%	34%	41%	53%	100%

Calculating D_{eff} for CSLB and PLSB using a minimum track length of 10, 15, and 20, there is a spread in D_{eff} (Fig. S2A). We conducted a one-way ANOVA to compare the means of the three groups for PSLB and CSLBs. We find for planar-supported bilayers, no significant statical difference between the three samples mean depending on track length ($F(2,1614)=0.27$, $p>0.05$) (Fig. S2B). However, for colloid-supported bilayers, the one-way ANOVA revealed a significant effect of the track length on the calculated D_{eff}, $F(2,1026)=20.70$, $p< 0.001$). Tukey post hoc test showed that there was a substantial significant difference between taking track length of 10 and 15, and for 10 and 20 ($p<0.001$).

We evaluated if there was any correlation between track length and $d\langle r^2\rangle/dt$ for planar- and colloid-supported bilayers (Fig. S2C). We initially evaluated Spearman correlation due to the large spread in data, with a monotonic relationship on the linear scale. We also performed a Pearson's correlation to avoid bias in our evaluation method. There was no significant correlation between track length and $d\langle r^2\rangle/dt$ for planar-supported bilayers using the first two data points ($P_{1\text{-}2}$). A slight negative Pearson correlation was observed between track length and $d\langle r^2\rangle/dt$ for colloid-supported bilayers, with this trend becoming much more evident on the log-log scale (Fig. S2C). We attributed the correlation between track length and $d\langle r^2\rangle/dt$ for colloids due to limitations in viewing only 1 μm diameter of the particle. In other words, if we think about a lipid traveling with a diffusion coefficient of 15 $\mu m^2 s^{-1}$, it will stay within the frame at a minimum track length of approximately 30 ms, in this case 3 frames. However, this issue does not occur for planar substrates, as the field of view we have imaged is 12x12 μm. This, of course, does not consider the random walk that lipid can have, increasing the probability of it staying within the field of view; however, it is a good indication as to why we should see a negative relationship for CSLB and not planar-supported bilayers and is an important imaging condition to consider. From this data evaluation, we decided to take the minimum track length of 10 for all data.

Imaging on a spherical surface imposed an upper limit on CSLB sptPALM. Lipids with D_{eff} >10 $\mu m^2/s$ moved out of focus before we could obtain sufficient track length for analysis, resulting in a slightly negative correlation between track length and D_{eff} (Fig. S2). This upper limit resulted in the underestimation of mean D_{eff}, making CSLB lipids appear slower and causing a confinement plateau in the MSD, which we termed the 3D boundary.

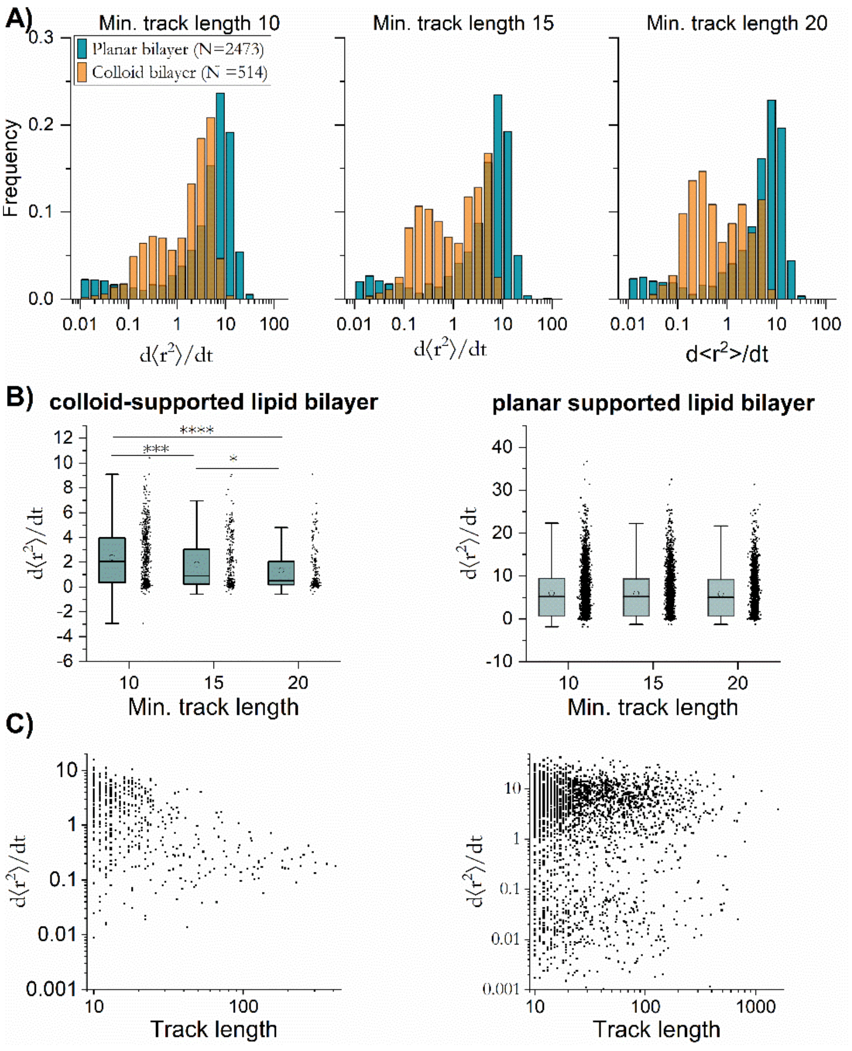

Figure S2. A) Distribution histogram of colloid and planar-supported DOPE-lipids $d\langle r^2\rangle/dt$ with minimum track length cuff-off of 10,15 and 20. **B)** Box Distribution of colloid (left) and planar (right) supported DOPE-lipids $d\langle r^2\rangle/dt$ with minimum track length cuff off of 10,15 and 20. With p<0.05=* and p<0.001=***. **C)** Correlation between interpolated $d\langle r^2\rangle/dt$ values and track length of colloid (left) and planar (right) supported DOPE-lipids $d\langle r^2\rangle/dt$

Next, we explored the influence of the minimum number of data points (either 2 or 3) for interpolation and their Y-offset (Fig. S3, Table S3). We examined taking position $P_{start\ point-end\ point}$. We found that the Y-offset increase for the starting point of 3 significantly differed from all other cases for CSLBs and PSLBs. For P_{3-4} and P_{3-6}, a significant increase in negative D values was observed, which are physically impossible and indicate inappropriate fitting. This finding aligns with the understanding that the data points with the most statistical information are data points 1 and 2. We discovered that there was not a significant difference in the interpolation mean values when taking two data points starting at either position 1 or 2 for either CSLBs or PSLBs; however, there appeared to be a slight increase in the number of negative values obtained. We evaluated the particle positional errors taken from the intercept of the fitting. With increasing starting point offset from 1 to 2, we observed a wider spread in the particle positional errors for colloid and planar samples. Therefore, we chose to analyze our data with P_{1-2}, accepting a relative error of approximately 41%. This error had insignificant effect on D_{eff} distribution and did not increase/decrease orders of magnitude.

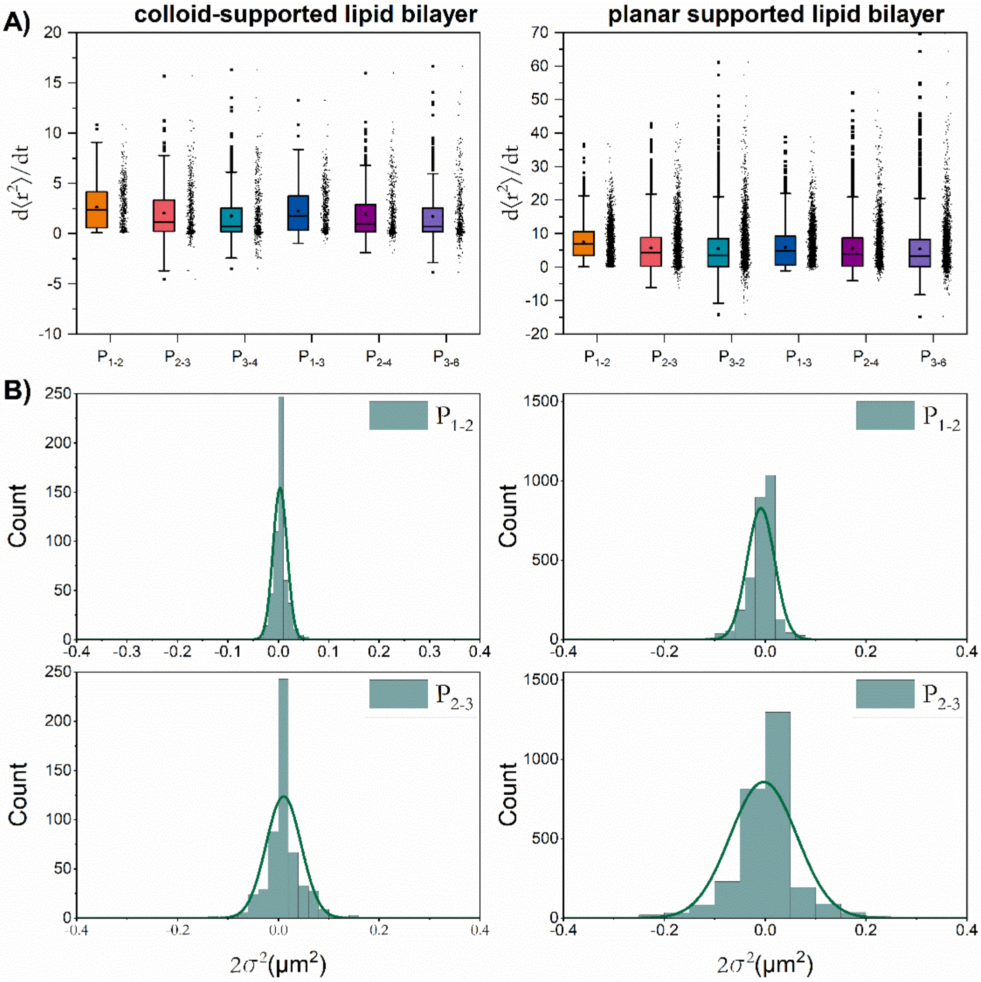

Figure S3. A) Distribution of colloid and planar supported DOPC bilayer interpolated data from d$\langle r^2 \rangle$/dt with varying the minimum number of points ($P_{start-end}$) with varying initial offsets (1,2,3) from the first non-zero data point. **B)** The Distribution of extrapolated particle positional error ($2s^2$) with P_{1-2} and P_{2-3} for colloid and planar-supported the DOPC bilayer. The minimum number of points in a track is 10, with N=539 for CSLB and N= 2841.

Table S2 Dunns multiple comparisons for CSLB and PSLB with a minimum track length of 10

**Colloid-supported lipid bilayers**					
	P_{2-3}	P_{3-4}	P_{1-3}	P_{2-4}	P_{3-6}
**P_{1-2}**	*0.0147	****<0.0001	ns	***0.0001	****<0.0001
**P_{2-3}**		*0.0345	ns	ns	**0.004
**P_{3-4}**			****<0.001	ns	ns
**P_{1-3}**				*0.0115	****<0.0001
**P_{2-4}**					ns
**Planar supported lipid bilayers**					
	P_{2-3}	P_{3-4}	P_{1-3}	P_{2-4}	P_{3-6}
**P_{1-2}**	**0.0021	****<0.0001	ns	****<0.0001	****<0.0001
**P_{2-3}**		*0.0105	ns	ns	*** 0.0002
**P_{3-4}**			****<0.001	ns	ns
**P_{1-3}**				**0.0047	****<0.0001
**P_{2-4}**					*0.0207

2. Quartile coefficient of dispersion

The quartile coefficient of dispersion (QCD) was used to evaluate the heterogeneity of samples due to being more robust to outliers.

$$QCD = \frac{Q3-Q1}{Q3+Q1} \quad \text{Equation S4}$$

3. RAW sptPALM particle tracks, MSD, and D_{eff} distribution of DOPC CSLBs

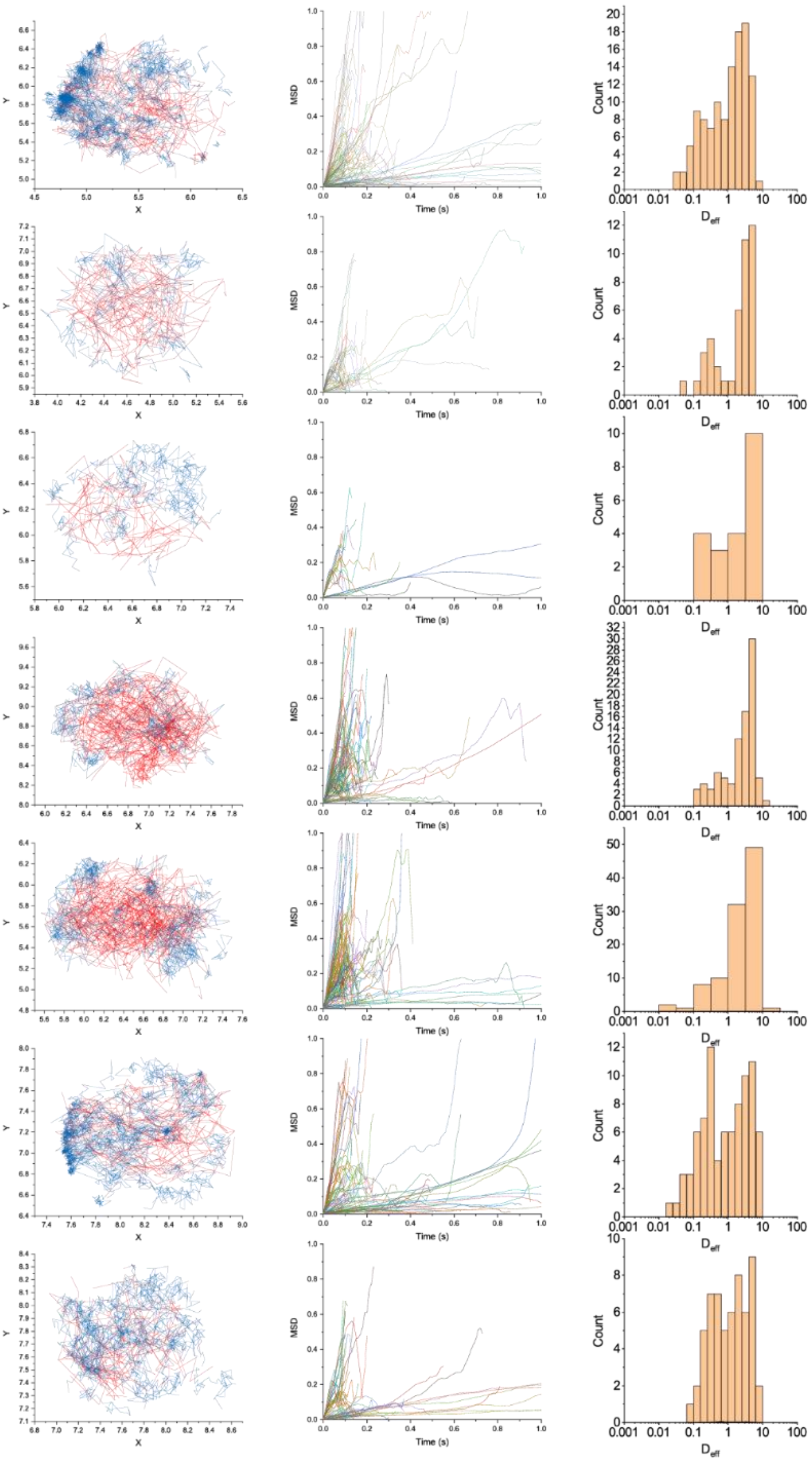

Figure S4. DOPC CSLBs all samples, with all **A)** measured tracks blue $D_{eff} < 1.78$, red $D_{eff} > 1.78$ μm²/s, **B)** MSD, and **C)** histogram of individual D_{eff}.

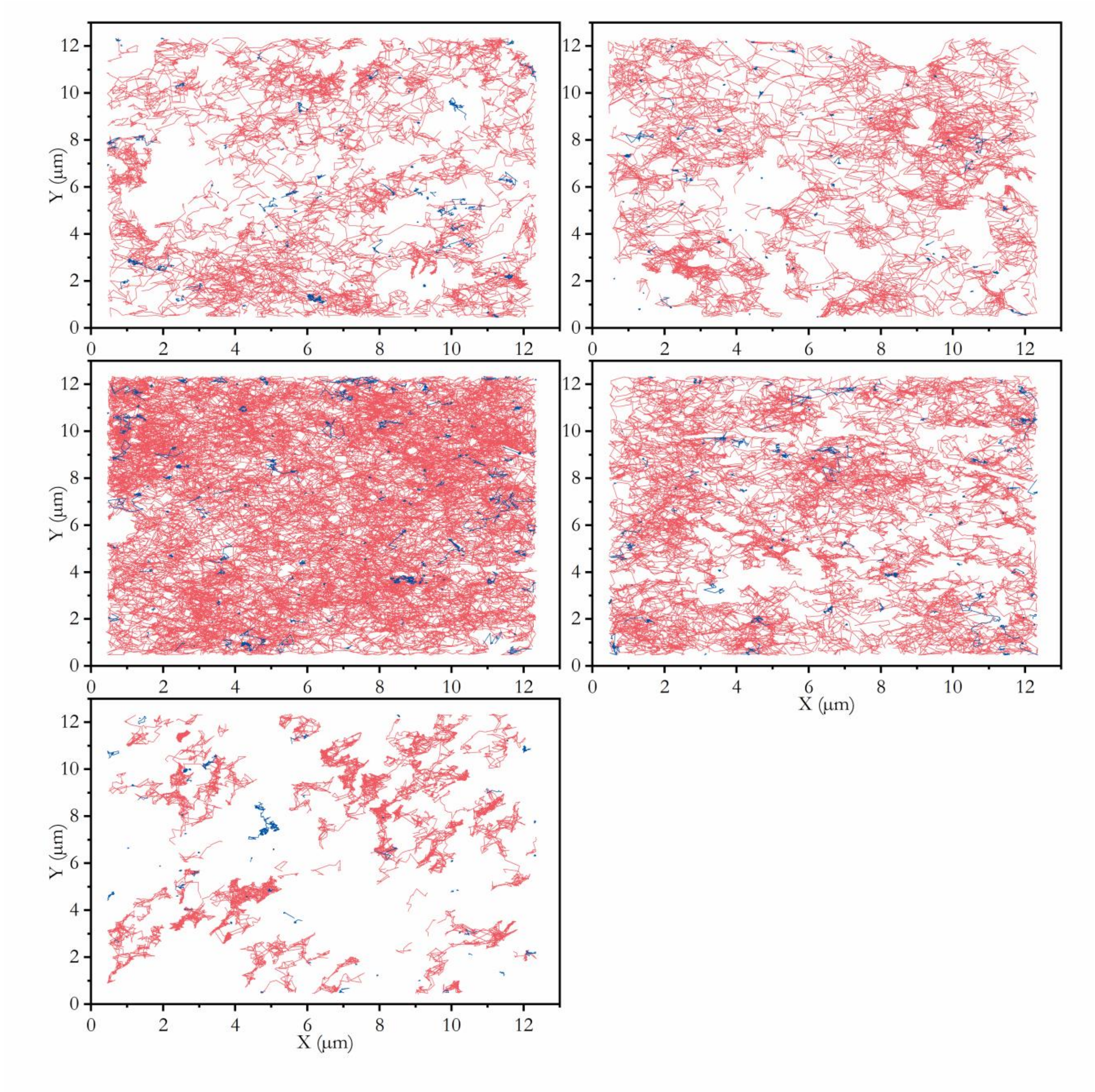

Figure S5. DOPC PSLBs all samples, with all measured tracks blue D_{eff} < 1.78, red D_{eff} > 1.78 µm^2/s

4. Confocal imaging and FRAP analysis

Confocal analysis was performed on Leica SP8 inverted microscope with a 100 x oil immersion objective (HC PL APO CS2 100x/1.40 OIL). Images (512 x 512 pixels) were recorded in the unidirectional scan mode at 600 Hz and a pinhole at 1 Airy unit (151 µm). Samples were excited with an argon laser at 488 nm, and emission signals were collected in 600–700 nm bands using PMT detector with 800 V gain.

Z-stacking from the bottom to top (by eye) of CSLB was performed to obtain 3D information. Step sizes were optimized via LAX software, and final images were displayed as the maximum intensity projections. For visualization, all images shown have been Z stacked via max projection, blurred, and background subtracted (Fig. S6).

The FRAP wizard LAS X Lecia software was used to perform FRAP experiments. Particles were imaged at the bottom at 5% laser intensity for a single frame. A region of interest of the particle was selected (0.5 x 0.5 µm) for bleaching for 5 frames at 0.866 seconds at 100% laser intensity. Post-bleaching samples were imaged for 20 frames at 0.866 seconds per frame, followed by 10 frames at 1 second per frame at the same laser intensity pre-bleach state (Fig. S7). Leica LAS software was used to analyze images and to generate FRAP recovery curves.

All fluorescence data were normalized ($f_{(t)}$) as described by Michul Kang[2] using Equation S5;

$$f_{(t)} = \frac{\left(\frac{ROI_{(t)} - Bkg_{(t)}}{Ref_{(t)} - Bkg_{(t)}}\right)}{\left(\frac{ROI_{(0)} - Bkg_{(0)}}{Ref_{(0)} - Bkg_{(0)}}\right)} \qquad \text{Equation S5}$$

where the ROI and Bkg are the fluorescence of an image's reference area and background, respectively. After normalization, the average of all samples was taken with a sample size of at least 9 particles.

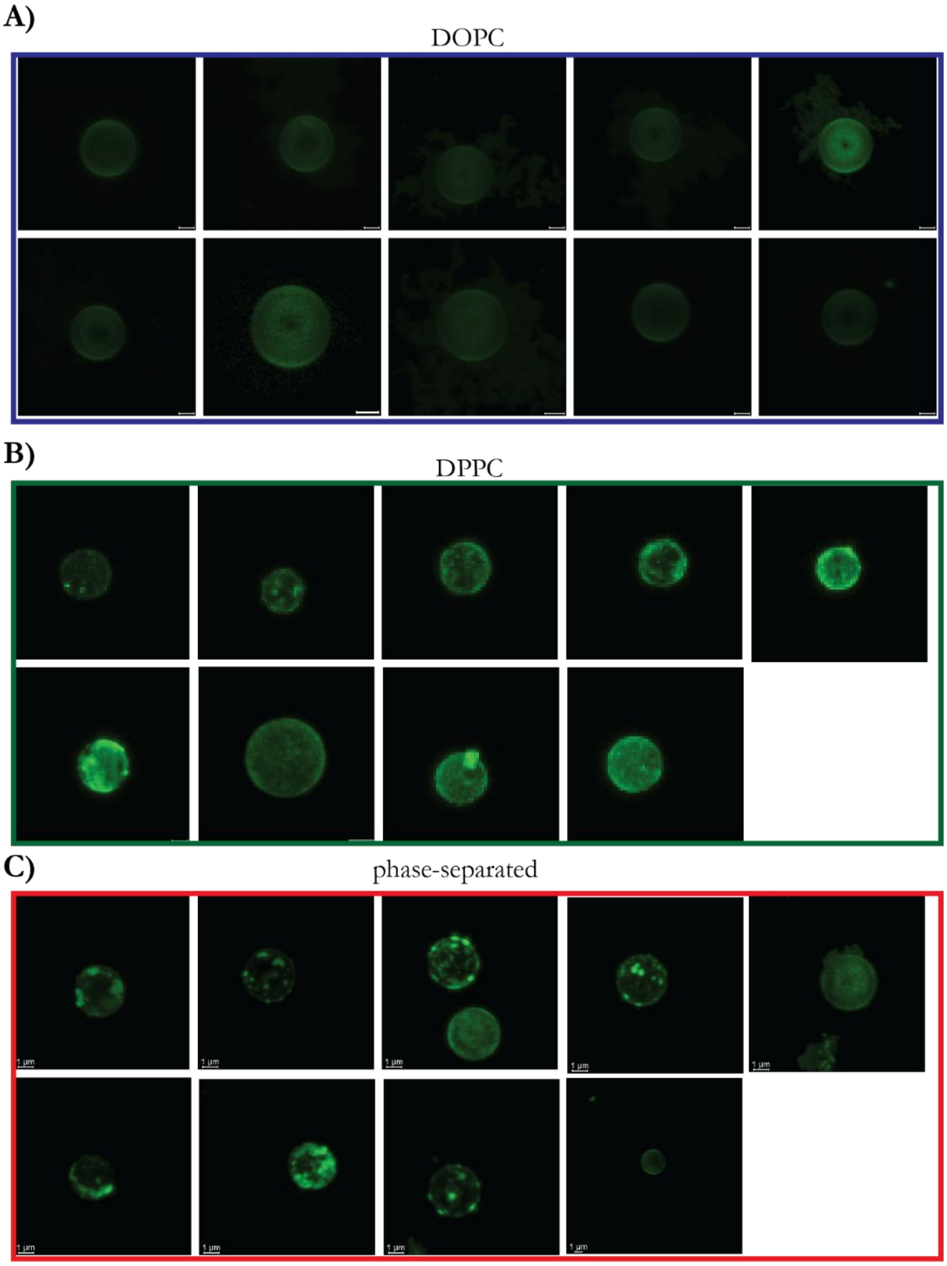

Figure S6 Maximum Z projection images of **A)** DOPC, **B)** DPPC, and **C)** phase-separated CSLBs.

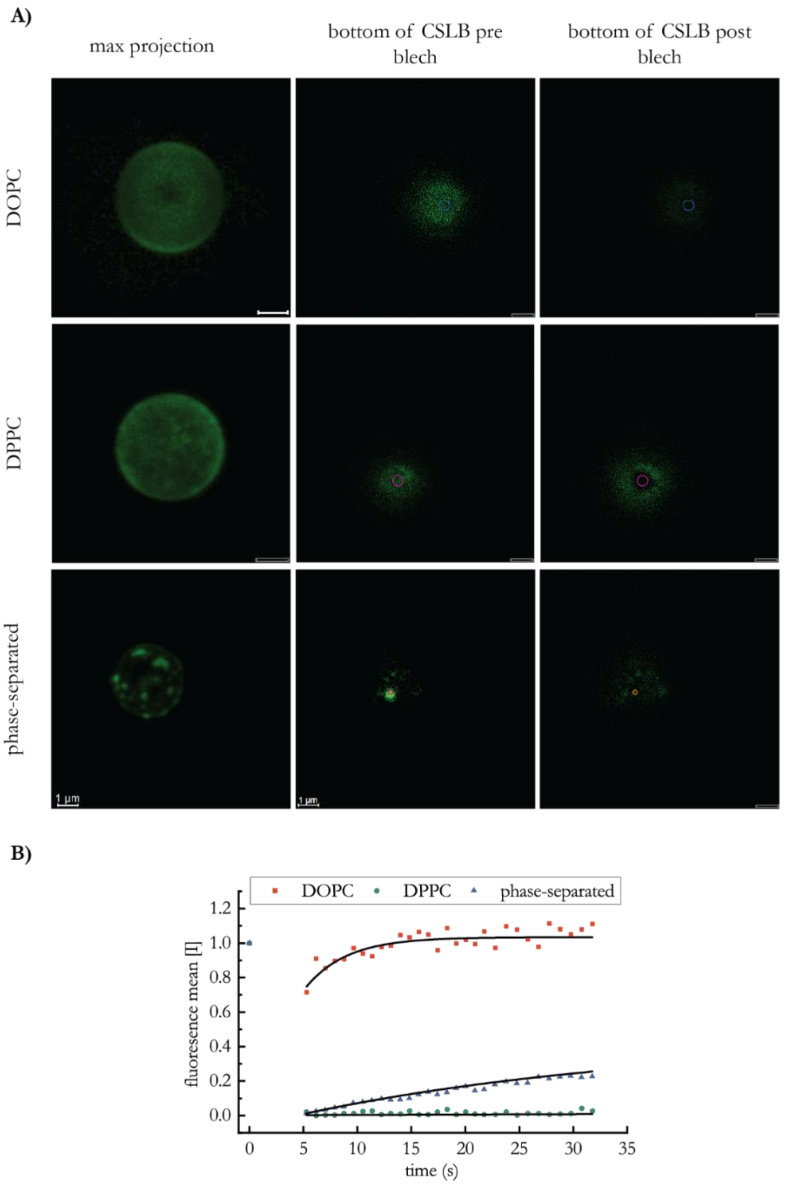

Figure S7 A) Examples of (left) z max projection taken of particles prior to bleaching, the (middle) bottom of particles prior to bleaching with ROI bleached, and (right) after bleaching **B)** FRAP recovery curve of DOPC, DPPC, and phase-separated CSLBs

5. References

1. Pinaud, F. *et al.* Dynamic Partitioning of a Glycosyl-Phosphatidylinositol-Anchored Protein in Glycosphingolipid-Rich Microdomains Imaged by Single-Quantum Dot Tracking. *Traffic* **10**, 691–712 (2009).
2. Kang, M., Day, C. A., Kenworthy, A. K. & DiBenedetto, E. Simplified equation to extract diffusion coefficients from confocal FRAP data. *Traffic* **13**, 1589–1600 (2012).
